# Broadband wide-angle terahertz antenna based on the application of transformation optics to a Luneburg lens

**DOI:** 10.1038/s41598-021-84849-8

**Published:** 2021-03-04

**Authors:** Yasith Amarasinghe, Rajind Mendis, Rabi Shrestha, Hichem Guerboukha, Jochen Taiber, Martin Koch, Daniel M. Mittleman

**Affiliations:** 1 School of Engineering, Brown University, Providence, RI 02912 USA; 2 Riverside Research, 2640 Hibiscus Way, Beavercreek, OH 45431 USA; 3 Philipps-Universität Marburg, Renthof 5, 35032 Marburg, Germany

**Keywords:** Electrical and electronic engineering, Applied optics

## Abstract

The design of antennas for terahertz systems remains a significant challenge. These antennas must provide very high gain to overcome significant free-space path loss, which limits their ability to broadcast or receive a beam over a wide angular range. To circumvent this limitation, here we describe a new device concept, based on the application of quasi-conformal transformation optics to the traditional Luneburg lens. This device offers the possibility for wide-angle beam steering and beam reception over a broad bandwidth, scalable to any frequency band in the THz range.

## Introduction

It has long been recognized that high-frequency communications links, operating above 100 GHz, will require high-gain antennas to overcome the large free-space path loss^1,2^. As a result, most researchers envision that terahertz wireless networks will employ highly directional, pencil-like beams, rather than omni-directional broadcasts that are commonly used in existing wireless systems. These terahertz systems will therefore require fast and agile beam steering. Of course, this could be accomplished using integrated phased arrays^3^. However, the implementation of integrated beam steering capabilities is still challenging in the terahertz range due mainly to the lack of practical phase shifters^4^. A variety of alternative solutions have been proposed, including mechanical systems^5,6^, liquid crystal devices^7^, and metasurfaces^8–10^.

Another possibility is enabled by the concepts of transformation optics^11,12^, which can be applied to well-known spherically symmetric lens designs to produce valuable new capabilities^13^. Here, we describe a modified Luneburg lens, in which the ray optics path from one facet of the lens to the other is converted from the conventional angle-to-angle mapping to an angle-to-position mapping^14^. This device can be used as a receiver with a wide angular aperture, when placed in front of a high-gain antenna such as a horn antenna. Alternatively, when used as a transmitter, small translations of the lens relative to a collimated input beam can be manifested as steering of an output beam over a large angular range. The device operates over a wide bandwidth of 50 GHz, and the concept is scalable to any broad frequency range in the millimeter-wave and terahertz spectrum. We describe the device design, based on quasi-conformal transformation optics (QCTO), and device fabrication using 3D printing. We characterize the device using THz time-domain spectroscopy, as well as in a wireless communication system test bed to verify that the device is compatible with high-data-rate signals.

## Design and fabrication

Our device design begins with the idea of a Luneburg lens, a gradient-index (GRIN) lens with a continuous spatial variation of the dielectric permittivity of the constituent material given by^15^:

1 $$\varepsilon_{r} = 2 - \left( {{\raise0.7ex\hbox{$r$} \!\mathord{\left/ {\vphantom {r R}}\right.\kern-\nulldelimiterspace} \!\lower0.7ex\hbox{$R$}}} \right)^{2} ,$$

where *r* is the radial distance from the center of the sphere, and *R* is the radius of the sphere. This well-known GRIN lens focuses a collimated input beam to the diametrically opposite point on the surface (that is, the input angle is mapped to the conjugate output angle). In our realization, we reduce the dimensionality of the lens from three to two, producing a cylindrically symmetric device in which radiation is confined in the third dimension by metal plates (i.e., a waveguide)^16^. In doing so, we exploit the fact that a parallel-plate metal waveguide, operated in the lowest-order transverse electric (TE_1_) mode, acts as a two-dimensional artificial dielectric in which the effective permittivity at a given frequency is determined by the spacing between the two metal plates^17,18^. By slowly varying the plate spacing (so that the plates are only quasi-parallel), we can realize an inhomogeneous artificial dielectric with nearly arbitrary permittivity variation^19^, which enables many examples of GRIN optics in the THz range. This idea still works if the metal waveguide is filled with a low-loss dielectric material, rather than air, although of course the effective permittivity profile is modified accordingly^20^.

**Figure 1:**
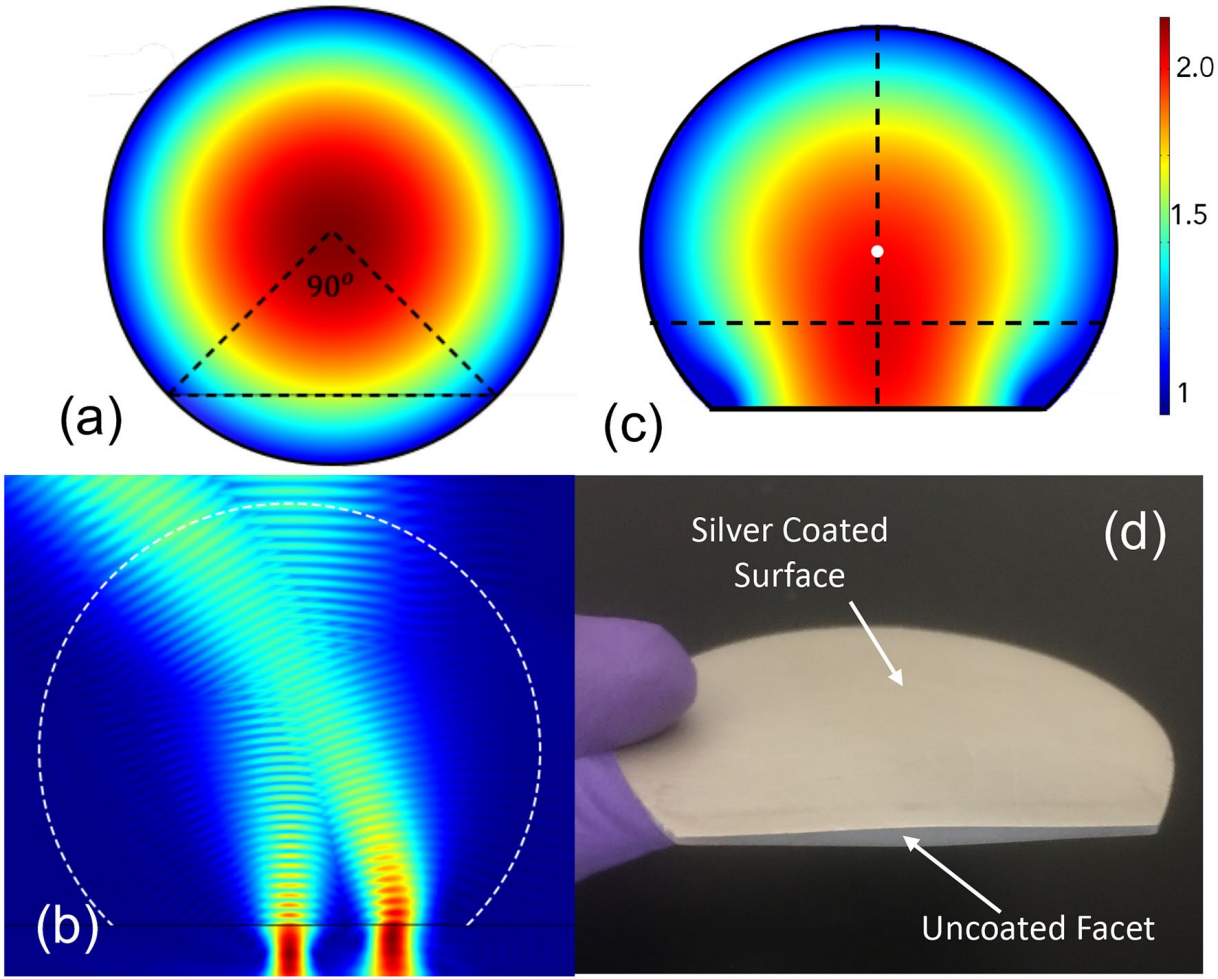
(**a**) Permittivity distribution of the original Luneburg lens, as defined by Eq. 1. (**b**) 2D numerical simulation of beam propagation in the device, with two different input beams entering through the top (curved) edge from two different angles produce a focused output beams that is normal to the flat (lower) surface. (**c**) Permittivity distribution of the transformed Luneburg lens. (**d**) Photo of one of the fabricated Luneburg lenses with silver coated on both top and bottom, with radiation shields removed.

Next, we use transformation optics to modify this (initially) cylindrically symmetric Luneburg lens. We designate a portion of the lens (inside the triangular region in Fig. 1a), and map this space (x, y, z) to the transformed space ($x^{\prime}$, $y^{\prime}$, $z^{\prime}$) according to

2 $$\varepsilon^{\prime} = \frac{{A\varepsilon A^{T} }}{det\left| A \right|} , \,\mu^{\prime} = \frac{{A\mu A^{T} }}{det\left| A \right|},$$

where $\varepsilon$ and $\mu$ are the original permittivity and permeability, $\varepsilon^{\prime}$ and $\mu^{\prime}$ are the transformed permittivity and permeability and A ($A_{ij} = \partial x_{i}^{^{\prime}} /\partial x_{j}$) is the Jacobian tensor which characterizes the coordinate transformation between the original and transformed spaces. This ‘flattens’ a portion of the lens, such that the initially curved edge becomes planar^13^. Using Dirichlet–Neumann (sliding) boundary conditions for the procedure guarantees that the transformed material is quasi-conformal^21^, which minimizes material anisotropy^22,23^. Similar ideas have been proposed for conformal transformations to minimize the anisotropy of the final design so that it can be realized in conventional dielectrics^24^, including modified Luneburg lenses for microwave^25^ and millimeter wave^26^ applications. Here, we extend these ideas to exploit the effective permittivity of the two-dimensional medium defined by the plate spacing of a quasi-parallel-plate metal waveguide.

We apply QCTO as described by Chang et al.^23^ to modify the geometry of the lens. The modified permittivity in the physical space is determined by solving Laplace’s equation using the procedure described in Biswas et al.^26^. The maximum permittivity resulting from the QCTO approach can be controlled by varying the angular range of the region of the lens that we want to flatten. In order to accommodate this and account for the dielectric material we use, we flatten a 90° section of the arc (Fig. 1a). As a result, an input beam entering through the curved (upper) part of the lens produces a focused output beam that is normal to the flat (lower) surface. This is illustrated in a 2D numerical simulation in Fig. 1(b), for two particular input beam angles (0° and 40°). The permittivity distribution profile matches with results from Biswas et al.^26^. This QCTO calculation allows us to determine the desired permittivity distribution ε(*x,y*) for the flattened Luneburg lens at a particular operating frequency *f*, as a function of position in the *xy* plane (as shown in Fig. 1c). We then use this continuously varying permittivity distribution ($\varepsilon \left( {x,y} \right)$) to define a non-uniform plate separation for a quasi-parallel plate waveguide, operating in the TE_1_ waveguide mode, according to:

3 $$b\left( {x,y} \right) = \frac{c}{{2f\sqrt {\varepsilon_{s} - \varepsilon \left( {x,y} \right)} }},$$

where *b*(*x,y*) is the plate separation at location (*x,y*), ε_s_ is the (assumed frequency-independent) dielectric constant of the material filling the space between the metal plates, and *f* is the chosen operating frequency. This approach assumes that the dielectric slab with thickness *b*(*x,y*) is coated on both surfaces with a perfect metal coating, and that the resulting waveguide operates in the TE_1_ mode. It also relies on the assumption that the spatial variation of the plate separation is slow enough so that no higher-order modes are excited in the interior of the waveguide due to abrupt changes in the plate spacing^18^.

Based on this calculation, we fabricate several devices in polystyrene using 3D printing, for two different operating frequencies: 150 GHz and 200 GHz. This material has ε_s_ ~ 2.48 in the frequency range of interest, which sets the upper limit of the permittivity range that can be achieved inside the waveguide, according to Eq. (3) (since ε(*x,y*) must be everywhere less than ε_s_). The 3D printer has a voxel resolution of 20 μm, corresponding to λ/75 for the higher of our two operating frequencies^27^. To further decrease the effects of roughness resulting from the 3D printing process, we use mechanical polishing to smooth out the surfaces of the device. After smoothing the surfaces, we apply silver paint to both surfaces of the polystyrene slab, to act as the two metal plates of the waveguide^16,20^. We also add metal foil as radiation shields on the top and bottom surfaces in order to block surface waves and scattered radiation. The fabricated lens is shown in Fig. 1d. The final device has a 25 mm curved radius and a 37.5 mm long flat surface.

## Spectral characterization

### Wide angle receiver

**Figure 2:**
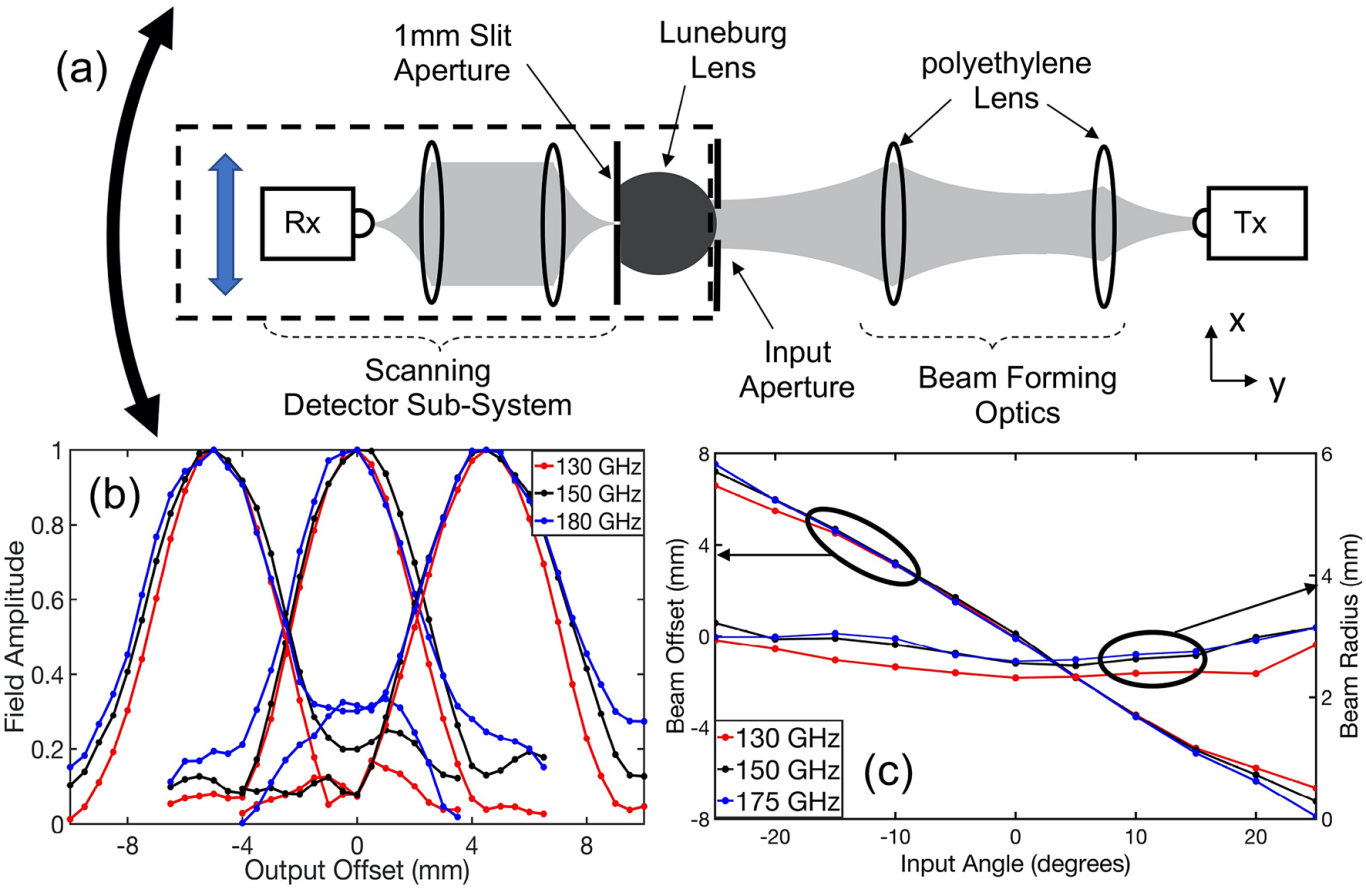
(**a**) Experimental setup for wide angle measurements. (**b**) Normalized electric field of the output beams at $+ 15^{ \circ }$, $0^{ \circ }$ and $- 15^{ \circ }$ for frequencies from 130 to 180 GHz. (**c**) Measured beam offset of the output beam and its radius as a function of the input beam angle.

Our first step in characterizing these modified Luneburg lenses is to measure their broadband frequency response using a THz time-domain spectrometer. In this section, we focus on the 150 GHz device. As shown in Fig. 2a, we use two confocal polyethylene lenses to form a frequency-independent input beam spot on the curved facet of the device. We situate a 10 mm diameter circular aperture in front of the device to control the input beam diameter. For this experiment we use a 10 mm diameter Gaussian beam at the input facet. The scanning detector subsystem also includes two confocal lenses, as well as a 1-mm slit aperture to improve the spatial resolution of the measurement. To excite the TE_1_ mode, the polarization of the incident THz beam is aligned parallel to the rim of the device [i.e., parallel to the lower (flat) metallized surface]. To characterize the output beam, we scan the detector subsystem along a line perpendicular to the optic axis (i.e., along the *x* direction) with a step size of 0.5 mm, ensuring that there is always a very small gap (< 0.05 mm) between flat edge of the device and the aperture. This is indicated by the vertical blue double arrow in Fig. 2a. We also carefully align the device axis with respect to the beam axis, so that the outgoing beam position is well defined relative to the input beam. In order to change the input angle, we rotate the device and scanning subsystem together (marked in black dashed box in Fig. 2a) with respect to the device center (shown as the white dot in Fig. 1c) and repeat the linear scan procedure. We repeat this for input angles varying from − 45° to + 45° in steps of 5°. This is indicated by the curved black double arrow in Fig. 2a.

The measured output beam profiles for three different input angles are shown in Fig. 2b, for three different frequencies. We observe that the output beam profiles remain the same for the whole frequency range of 130–180 GHz (a bandwidth 50 GHz). Figure 2c shows the lateral displacement of the output beam and the beam size as a function of the input beam angle. In this figure, we compute the beam size by measuring the half-width-at-half-maximum beam radius. We see that the output beam’s center is displaced linearly as a function of input beam angle, with little change in the beam size. This demonstrates near-ideal mapping between input angle and output position, over an input angular range of about 50°, with only minor variations near the edges of the operational range due to minor imperfections in the device geometry. In these plots, the offset of the output beam is always calculated relative to the device axis. We also observe that the output beam power varies by less than 2 dB over the entire range of input angles, indicating that the insertion loss is nearly independent of angle.

### Steerable transmitter

**Figure 3:**
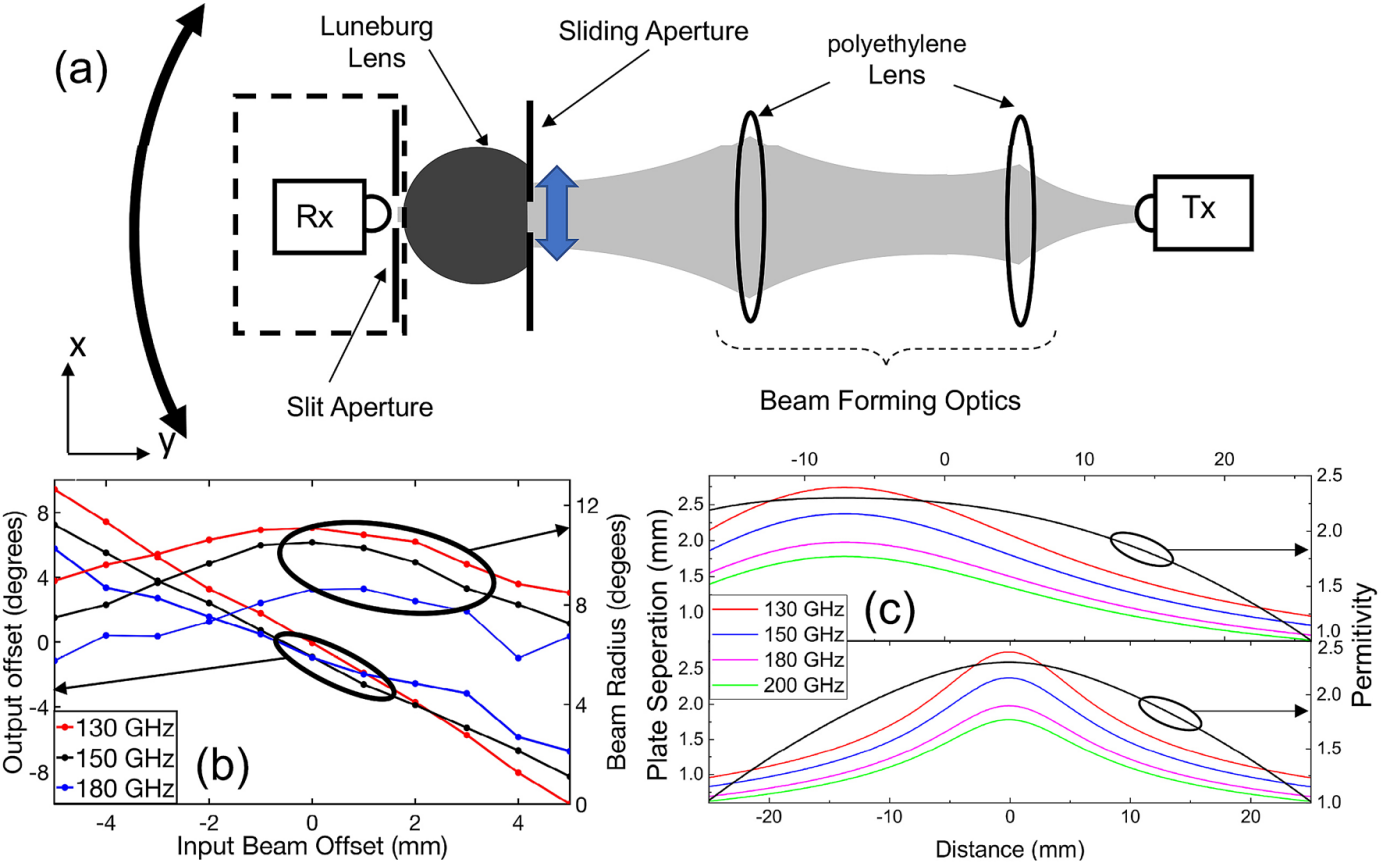
(**a**) Experimental setup for beam steering application. (**b**) Measured beam offset of the output beam and its radius as a function of the input beam offset. (**c**) The plate separation and the permittivity profiles of an ideal device, at several different frequencies, along two different cross-sections indicated by dashed lines in Fig. 1b. These are computed using Eq. (3). In these figures, the black solid curves give the desired permittivity profiles along the same two cross-sections.

Next, we flip the device around, to explore its use for beam steering. In this case, the input beam is directed at the flat facet of the lens, rather than the curved face. The measurement setup (Fig. 3a) is similar to that used earlier. We insert a 5 mm circular aperture in front of the flat facet, in order to control the size and position of the region of this input face that is illuminated by the incoming beam. We measure the output beam by rotating the detector subsystem (with respect to device center), which consists of a 4 mm circular aperture and detector, situated very close to the lens as described above. We characterize this output signal for many different positions of the input slit aperture. For this experiment we used an angular step size of 1°. Both the output offset angle and input aperture position are measured relative to the device axis, which once again is coincident with the beam axis.

The results, shown in Fig. 3b, once again confirm the near-ideal performance of the device. The output beam scans through an angular range of about 16°, varying linearly with the location of the input illumination spot. Meanwhile, the output beam’s size is nearly invariant over this entire angular scanning range. The reason that the output beam steering range (16°) is smaller than the input angular acceptance range observed in Fig. 2 (25°) is because of an asymmetry in the coupling efficiency from free space into the device, as well as the finite size of the input beam. Near the edges of the device along the flat facet, the plate separation is smaller, so the input coupling efficiency (for a fixed input beam size) decreases, and the amplitude of the (15 mm diameter) Gaussian beam is also smaller near the edges.

We note that even though this device was designed to operate at a single frequency (the design frequency of 150 GHz), the experimental results confirm that the device actually operates over a relatively broad bandwidth of 50 GHz. We can understand this by examining the plate separation profiles (i.e., the predictions of Eq. 3) for several different frequencies. These are plotted in Fig. 3c for the two cross sections shown as dotted lines in Fig. 1c. We observe that, over a wide range of frequencies, the plate spacing profiles follow the same curve with a small offset from each other. It is therefore not surprising that the device maintains approximately similar functionality over a reasonably broad (50 GHz) band centered on the design frequency. Based on similar measurements on the 200 GHz device, we also observe a near-ideal performance over a bandwidth of approximately 50 GHz.

## Characterization using a communication signal

### Power measurements

**Figure 4:**
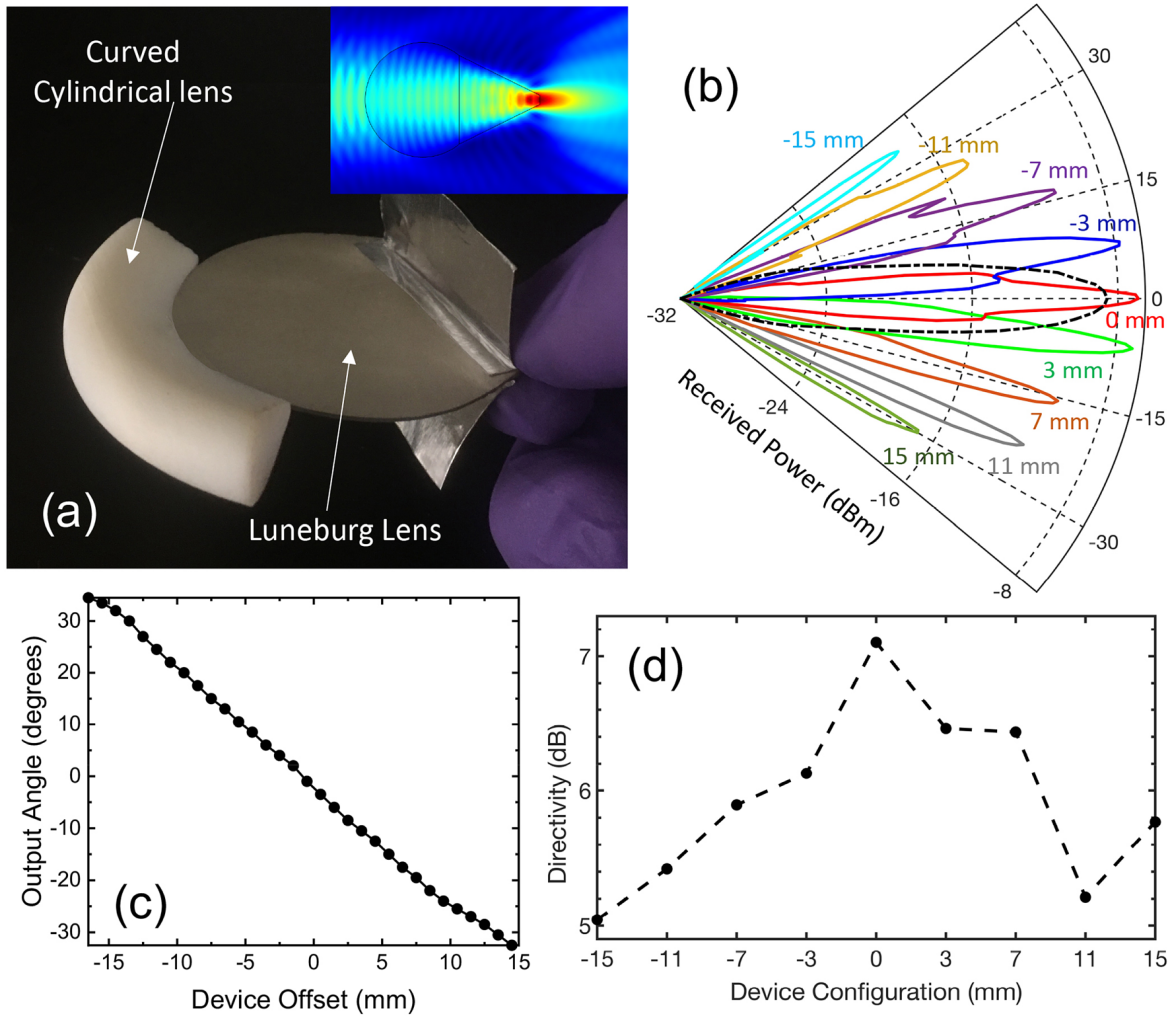
(**a**) Photograph of the device with curved cylindrical lens attached. (inset: 2D numerical simulation of beam focusing using the cylindrical lens). (**b**) Output beam profiles in a polar plot. (**c**) Measured output beam angle as a function of the device offset. (**d**) Directivity of the device extracted from our measurements shown in (**b**).

After confirming the beam steering and reception application of the device using broadband characterization, we next characterize the devices using a single-frequency carrier wave, modulated with a digital data stream. In this case, we use the Luneburg lens that was designed using the same procedure as above except with a different design frequency, shifted to 200 GHz to accommodate the frequency of our communications test bed. This test bed employs a frequency multiplier chain (FMC), producing a modulated signal (on–off keying) at a carrier frequency of 200 GHz with a data rate of 1.1 Gbit/s. We detect these signals using a zero-bias Schottky diode as a receiver, and demodulate the signal for bit-error-rate measurements. We note that, at this higher frequency, the plate separation of the waveguide shrinks to approximately 0.6 mm on the curved facet of the lens. A 200 GHz beam emerging through such a small facet would experience significant diffraction in the direction perpendicular to the plane of the lens, and would spread so rapidly that it would be essentially useless for communications at any reasonable range. In order to circumvent this issue, we design an add-on to the Luneburg lens which is essentially a cylindrical lens that attaches to the curved input facet of the device. This consists of a part of a cylinder of 1 cm diameter, attached to a symmetrical trapezoidal section (with 7.5 mm height) to mate the hyper-hemicylinder to the Luneburg lens. The bases of this trapezoid are 0.6 mm (on the side connecting to the Luneburg lens) and 7.5 mm (on the side connecting to the hemi-cylinder). In order to have a smooth transition from the cylinder part to the trapezoidal part, we extend the half cylinder from the center by 2.5 mm to the flat surface. In this configuration the hyper-hemicylinder has 7.5 mm long flat surface and the size of this hyper-hemicylinder is 7.5 mm (inset to Fig. 4a). This entire structure is curved along the axis perpendicular to the beam propagation direction, so that it fits onto the curved face of the Luneburg lens. The entire structure (Luneburg lens + trapezoidal mating + hyper-hemicylinder) is fabricated monolithically using 3D printing (see Fig. 4a). This device enhancement (shown in Fig. 4a), also fabricated using polystyrene 3D printing, dramatically reduces the effects of diffraction for the beam emerging from the subwavelength aperture, as shown in the inset to Fig. 4a. We also note that this enhancement improves the collection efficiency, if the device is used as a receiver (rather than a transmitter, as considered here), due to the larger (1 cm) aperture.

With this modified Luneburg lens, we repeat the experiments illustrated in Fig. 3a, except now with the modulated carrier wave. The Luneburg lens is placed directly in front of the horn antenna that couples the FMC signal into free space. To excite the TE_1_ mode, the polarization of the incident THz beam from the horn antenna is aligned parallel to the rim of the device, which is situated in the center of the horn antenna. We characterize the far-field radiation pattern and bit error rate as a function of angle, by moving the receiver along an arc at a distance of 37 cm from the transmitter horn antenna. We then shift the Luneburg lens to a slightly offset position, away from the center of the transmitter horn antenna, and repeat the angular characterization. In this fashion, we can characterize the far-field output pattern for all possible input excitation positions on the flat surface of the lens. The results, shown in Fig. 4b, demonstrate wide-angle beam steering over a range of more than 70°, arising from only about 2.6 cm of lateral translation of the lens. In this polar plot the power is displayed in dBm, with – 32 dBm as the noise floor. The relation between output angle and input position is again linear, as shown in Fig. 4c (and as expected from the results of Fig. 3). We also measure the beam emerging directly from the horn antenna, without the Luneburg lens in place. This result (dashed curve in Fig. 4b) shows that the lens narrows the beam pattern, compared to the beam at its input facet, providing additional directivity gain. In order to have better understanding of the device performance, we extract the directivity of the device from the measurements shown in Fig. 4b. This result is plotted in Fig. 4d on a dB scale, relative to the value of the directivity measured without the device in the beam path (i.e., just the horn antenna). We find that the highest directivity is obtained when the device has no lateral displacement, but it only varies by about 2 dB for any position of the device. Compared to the bare horn antenna, we find a directivity gain of around 6 dB on average.

### Bit error rate measurements

**Figure 5:**
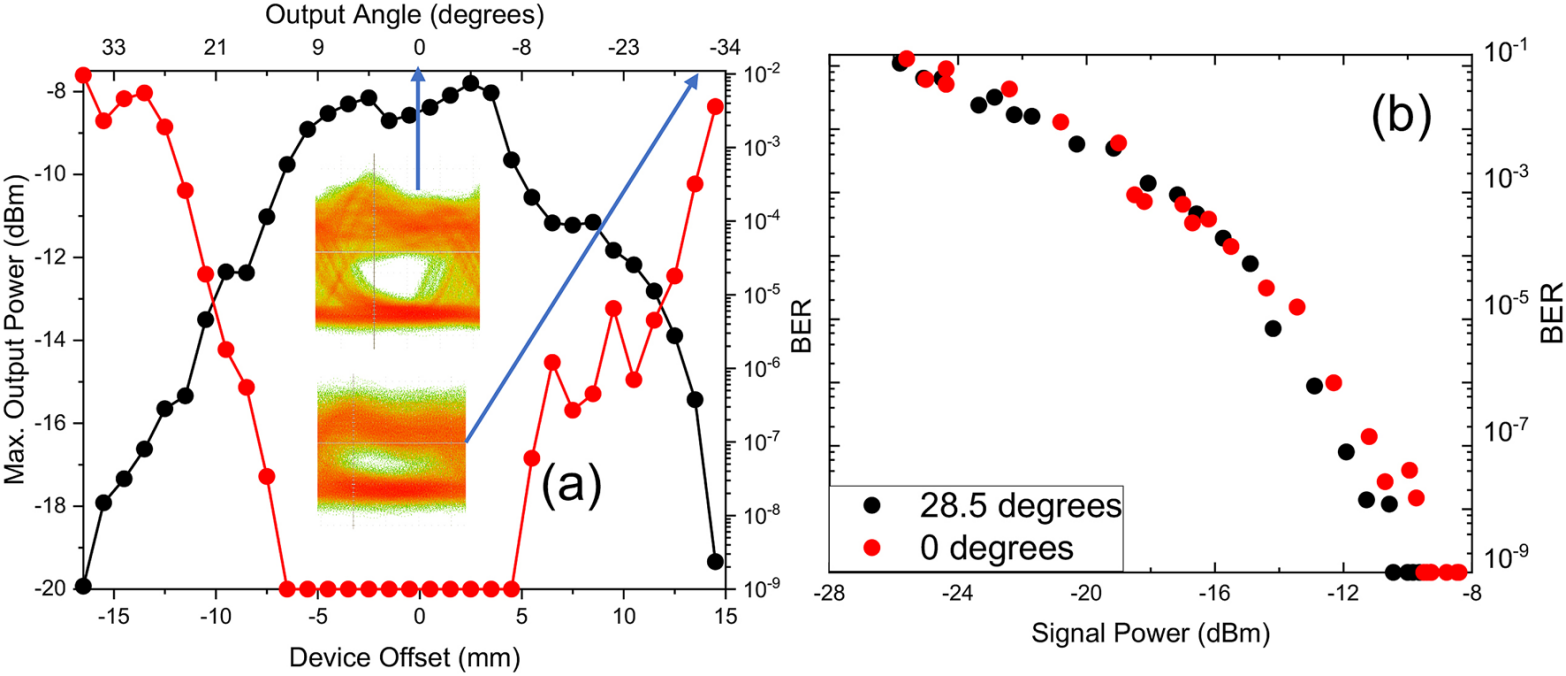
(**a**) Received power (black dots) and BER (red dots) corresponding to that power, as a function of device offset and output angle. (inset: eye diagrams at 0 mm and 14 mm device offset) (**b**) BER as a function of received signal power for two different input angles. The BER tester used in these measurements saturates at 10^–9^.

Finally, in order to verify the data transmission capabilities of the device, we characterize the performance using modulated data and bit error rate (BER) testing. Figure 5a shows the received power and the corresponding BER as a function of the device offset, for a fixed input power. We see that the received power drops when the device moves away from the input beam axis. This can be understood by examining the plate separation profile shown in Fig. 3b. The power coupled into the device reduces with the device offset from the beam axis because the plate separation decreases while in our measurements the input beam spot size remains constant. This leads to a degradation of the BER. Measured eye diagrams, taken at offsets of zero and 15 mm, illustrate this expected behavior when the coupled power (and therefore the detection SNR) decreases. To verify that this change in BER performance is due only to decreased input power coupling efficiency, we measure the BER as a function of the received signal power for two angles (Fig. 5b), one coinciding with the beam center axis (red dots) and the other with the edge of the device operation range (black dots). There is no observable difference between the power dependence of these two BER curves. This indicates that the data transmission characteristics are not influenced by the output angle; only the power coupling efficiency changes. Thus, we conclude that effects such as the dispersion of the waveguide do not contribute significantly to the BER at this modulation rate. Increasing the transmitter power would be an effective strategy to offset the decreasing power coupling at higher offsets.

## Conclusion

We have designed and tested a flattened Luneburg lens for the THz regime, based on the application of quasi-conformal transformation optics. This device offers the possibility for wide-angle beam steering and beam reception over a broad bandwidth, scalable to any frequency band in the THz range. Specifically, this device can accommodate a beam in a 2D plane within a total collection angle of 50° in the frequency range from 130 to 180 GHz. We demonstrate that we can use this device to steer a beam simply by shifting the feeder position by small amounts, along the input facet of the lens. We also verify that this device is compatible with high-data rate transmissions, establishing its feasibility for use in future wireless communication systems.

## References

[1] Sengupta K, Nagatsuma T, Mittleman DM (2018). Terahertz integrated electronic and hybrid electronic–photonic systems. Nat. Electron..

[2] Piesiewicz R (2007). Short-range ultra-broadband terahertz communications: Concepts and perspectives. IEEE Antennas Propag. Mag..

[3] Sengupta K, Hajimiri A (2012). A 0.28 THz power-generation and beam-steering array in CMOS based on distributed active radiators. IEEE J. Solid-State Circuits.

[4] Headland H, Monnai Y, Abbott D, Fumeaux D, Withayachumnankul W (2018). Tutorial: Terahertz beamforming, from concepts to realizations. APL Photon..

[5] Monnai Y (2013). Terahertz beam steering and variable focusing using programmable diffraction gratings. Opt. Express.

[6] Seifert JM, Hernandez-Cardoso GG, Koch M, Castro-Camus E (2020). Terahertz beam steering using active diffraction grating fabricated by 3D printing. Opt. Express.

[7] Altmann K (2013). Polymer stabilized liquid crystal phase shifter for terahertz waves. Opt. Express.

[8] Chen P, Argyropoulos C, Alù A (2013). Terahertz antenna phase shifters using integrally-gated graphene transmission lines. IEEE Trans. Antennas Propag..

[9] Zhang Y (2019). Large phase modulation of THz wave via an enhanced resonant active HEMT metasurface. Nanophotonics.

[10] Chen H (2009). A metamaterial solid-state terahertz phase modulator. Nat. Photon..

[11] Pendry JB, Schurig D, Smith DR (2006). Controlling electromagnetic fields. Science.

[12] Leonhardt U (2006). Optical conformal mapping. Science.

[13] Kundtz N, Smith DR (2010). Extreme-angle broadband metamaterial lens. Nat. Mater..

[14] Ok G, Park K, Kim HJ, Chun HS, Choi SW (2014). High-speed terahertz imaging toward food quality inspection. Appl. Opt..

[15] Luneburg R (1944). Mathematical Theory of Optics.

[16] Liu J, Mendis R, Mittleman DM (2013). A Maxwell's fish eye lens for the terahertz region. Appl. Phys. Lett..

[17] Mendis R, Mittleman DM (2010). A 2-D artificial dielectric with 0 ≤ n < 1 for the Terahertz region. IEEE Trans. Microw. Theory Tech..

[18] Mendis R, Nagai M, Wang Y, Karl N, Mittleman DM (2016). Terahertz artificial dielectric lens. Sci. Rep..

[19] Mendis R, Liu J, Mittleman DM (2012). Terahertz mirage: Deflecting terahertz beams in an inhomogeneous artificial dielectric based on a parallel-plate waveguide. Appl. Phys. Lett..

[20] Amarasinghe Y, Mittleman DM, Mendis R (2019). A Luneburg lens for the Terahertz region. J. Inf. Milli. THz Waves.

[21] Thompson JF, Sdoni BK, Weatherill NP (1999). Handbook of Grid Generation.

[22] Li J, Pendry JB (2008). Hiding under the carpet: A new strategy for cloaking. Phys. Rev. Lett..

[23] Chang Z, Zhou X, Hu J, Hu G (2010). Design method for quasi-isotropic transformation materials based on inverse Laplace’s equation with sliding boundaries. Opt. Express.

[24] Landy NI, Padilla WJ (2009). Guiding light with conformal transformations. Opt. Express.

[25] Demetriadou A, Hao Y (2011). Slim Luneburg lens for antenna applications. Opt. Express.

[26] Biswas S (2019). Realization of modified Luneburg lens antenna using quasi-conformal transformation optics and additive manufacturing. Microwave Opt. Technol. Lett..

[27] Guo R (2019). 3D printed Terahertz rectangular waveguides of polystyrene and TOPAS: A comparison. J. Infrared Milli. THz Waves.

